# Ecotin-like serine peptidase inhibitor ISP1 of *Leishmania major* plays a role in flagellar pocket dynamics and promastigote differentiation

**DOI:** 10.1111/j.1462-5822.2012.01798.x

**Published:** 2012-05-08

**Authors:** Lesley S Morrison, Amy Goundry, Marilia S Faria, Laurence Tetley, Sylvain C Eschenlauer, Gareth D Westrop, Anna Dostalova, Petr Volf, Graham H Coombs, Ana Paula C A Lima, Jeremy C Mottram

**Affiliations:** 1 Wellcome Trust Centre for Molecular Parasitology, Institute of Infection, Immunity and Inflammation, College of Medical, Veterinary and Life Sciences, University of GlasgowGlasgow G12 8TA, UK; 2 Instituto de Biofisica Carlos Chagas Filho, Universidade Federal do Rio de JaneiroRio de Janeiro, RJ, 21949-900, Brazil; 3 Strathclyde Institute of Pharmacy and Biomedical Sciences, University of StrathclydeGlasgow G4 0RE, UK; 4 Charles University in Prague, Faculty of Science, Dep. ParasitologyVinicna 7, Prague 2, CZ 128 44, Czech Republic

## Abstract

*Leishmania* ISPs are ecotin-like natural peptide inhibitors of trypsin-family serine peptidases, enzymes that are absent from the *Leishmania* genome. This led to the proposal that ISPs inhibit host serine peptidases and we have recently shown that ISP2 inhibits neutrophil elastase, thereby enhancing parasite survival in murine macrophages. In this study we show that ISP1 has less serine peptidase inhibitory activity than ISP2, and in promastigotes both are generally located in the cytosol and along the flagellum. However, in haptomonad promastigotes there is a prominent accumulation of ISP1 and ISP2 in the hemidesmosome and for ISP2 on the cell surface. An *L. major* mutant deficient in all three *ISP* genes (Δ*isp1/2/3*) was generated and compared with Δ*isp2/3* mutants to elucidate the physiological role of ISP1. In *in vitro* cultures, the Δ*isp1/2/3* mutant contained more haptomonad, nectomonad and leptomonad promastigotes with elongated flagella and reduced motility compared with Δ*isp2/3* populations, moreover it was characterized by very high levels of release of exosome-like vesicles from the flagellar pocket. These data suggest that ISP1 has a primary role in flagellar homeostasis, disruption of which affects differentiation and flagellar pocket dynamics.

## Introduction

The leishmaniases are an array of diseases ranging symptomatically from relatively mild, local, cutaneous ulceration to fatal, visceral dissemination with accompanying fever and anaemia (Murray *et al*., 2005). They are caused by the protozoan parasites of the *Leishmania* genus and are transmitted by phlebotomine sand flies. *Leishmania* are digenetic parasites that alternate between disease-causing, non-motile, intracellular amastigotes and flagellated, extracellular promastigotes in their mammalian hosts and insect vectors respectively. *Leishmania* amastigotes are ingested by sand flies in a blood meal and undergo serial differentiation before they are injected, as metacyclic promastigotes, into their subsequent mammalian host. The intermediate stages in differentiation from procyclic to metacyclic promastigote are often overlooked as they are seldom seen *in vitro* (Rogers *et al*., 2002; Gossage *et al*., 2003). Nevertheless the distinct phenotypes afforded during these intermediate life cycle stages are critical for the survival of the parasite, establishment of infection and transmission to the next host. Procyclic promastigotes differentiate from amastigotes in the blood meal surrounded by the peritrophic matrix (PM), where they rapidly multiply. Simultaneously with the end of blood meal digestion and disintegration of the PM, procyclic promastigotes transform into nectomonads (Sadlova and Volf, 2009), long, non-replicative forms that escape from the remains of the PM-encased blood meal and specifically attach to the midgut epithelium to prevent expulsion from thegut during defecation (Wilson *et al*., 2010). Here they develop into leptomonad promastigotes (a synonym for short nectomonads) (Cihakova and Volf, 1997) which enter another proliferative cycle and finally transform into the mammalian infective stage, metacyclic promastigotes (Rogers *et al*., 2002; Gossage *et al*., 2003). Leptomonads and metacyclics colonize the stomodeal valve (junction between midgut and foregut) while tear-shaped haptomonads adhere to the stomodeal valve (Molyneux and Killick-Kendrick, 1987). Ultimately, the valve is blocked by accumulated parasites and promastigote secretory gel (Rogers *et al*., 2002), and its chitin lining is destroyed by *Leishmania* chitinase (Volf *et al*., 2004; Rogers *et al*., 2008). These changes facilitate transmission of the infective metacyclic promastigotes to the mammalian host (Rogers *et al*., 2004).

*Leishmania* amastigotes are often erroneously referred to as aflagellate however they have a short internal flagellum that extends upon differentiation into procyclic promastigotes (Gluenz *et al*., 2010a). The promastigote flagellum consists of the canonical 9+2 microtubules, motile axoneme and an associated paraflagellar rod (PFR), which is also critical for motility (Santrich *et al*., 1997). Flagellum length is dynamic during the various promastigote stages of the parasite's life cycle, where it is crucial for directed motility and attachment within the sand fly. It varies from a relatively short, < 7 µm, in procyclic promastigotes, to in excess of 20 µm in the colonizing nectomonad and leptomonad promastigotes, while adherent haptomonads have reduced flagella with an extended tip (Killick-Kendrick *et al*., 1974b; Rogers *et al*., 2002). Eukaryotic flagella are assembled/disassembled using an intraflagellar transport (IFT) system. IFT in *Leishmania* has not been widely studied but an *in silico* genome screen has shown that the IFT pathway is present (Gouveia *et al*., 2007) and it has been investigated in the closely related *Trypanosoma brucei,* where silencing of either anterograde or retrograde transport results in shortening of the flagellum (Absalon *et al*., 2008). Variations in *Leishmania* flagellum length may also be dictated by IFT-independent mechanisms affecting axonemal microtubule dynamics and protein trafficking (Gluenz *et al*., 2010b). While motility and attachment are clearly of vital importance in promastigotes, accumulating evidence suggests that the flagellum also functions as a sensory organelle promoting environmentally induced morphological changes during parasite differentiation (Zilberstein and Shapira, 1994; Rotureau *et al*., 2009) and amastigote–host interactions (Gluenz *et al*., 2010a).

The *Leishmania* flagellum exits the cell body from an invagination in the cell membrane that forms the flagellar pocket, the parasite's sole site for endo/exocytosis (Field and Carrington, 2009). However, while the parasite assembles and routes most surface molecules, such as lipophosphoglycan and GP63, via the classical ER-Golgi-plasma membrane pathway (McConville *et al*., 2002), it is recognized that many *Leishmania-*secreted proteins have no N-terminal secretion signal indicating that the parasite employs non-classical methods of secretion (Stegmayer *et al*., 2005). Recent evidence suggests that more than 50% of *Leishmania*-secreted proteins are trafficked in vesicles morphologically similar to mammalian exosomes (Silverman *et al*., 2010); but while there is accumulating evidence for the stimulated secretion of exosomes and shedding vesicles in macrophages and neutrophils (Cocucci *et al*., 2009) the mechanism for vesicle-mediated non-classical secretion by *Leishmania* is unknown.

*Leishmania* has three genes encoding ecotin-like inhibitors of serine peptidases (ISPs) (Eschenlauer *et al*., 2009). Ecotin from *Escherichia coli* is a strong competitive inhibitor of trypsin-fold serine peptidases (Chung *et al*., 1983) and its postulated *in vivo* targets include mammalian serine peptidases, such as neutrophil elastase (NE), tryptase and cathepsin G, expressed by cells of the innate immune system (Eggers *et al*., 2004). Trypsin-fold serine peptidases of clan PA, family S1A are not apparently encoded in the *Leishmania* genome (Ivens *et al*., 2005), so we hypothesized that host-derived serine peptidases are the major target for parasite ISPs. ISP2 is expressed in all life cycle stages of *Leishmania major* with particularly high levels in infective metacyclic promastigotes and amastigotes, where it influences the early stages of macrophage infection and parasite intracellular survival through inhibition of NE present at the surface of macrophages (Eschenlauer *et al*., 2009; Faria *et al*., 2011). *L. major* lines lacking *ISP*2 and *ISP*3 (Δ*isp*2/3) are internalized more efficiently than wild-type (WT) parasites by BALB/c or C57B6 macrophages, due to upregulation of phagocytosis and selective engagement of CR3 (Eschenlauer *et al*., 2009; Faria *et al.*, 2011). However, deficiency in ISP2 and ISP3 results in the partial elimination of intracellular parasites 24 h post-infection, suggesting that these ISPs are important for the initiation and persistence of infection in the mammalian host. NE is the main target of ISP2 in macrophages and NE activity, subject to control by ISP2, promotes the phagocytosis of *L. major* through Toll-like receptor 4 (TLR4) and provokes the killing of a proportion of the internalized parasites within 24 h (Faria *et al*., 2011). These studies indicate that ISP2 regulates host serine peptidase activity, which influences the outcome of anti-parasite defences.

In this study we created an *L. major* mutant deficient in all three ISP proteins (Δ*isp1/2/3*). By comparing the phenotype of Δ*isp2/3 and*Δ*isp1/2/3* mutants we have been able to define distinct roles for ISP1 and ISP2 in *Leishmania*.

## Results

### Generation of *L. major* ISP1, ISP2 and ISP3 triple null mutants

**Fig. 1:**
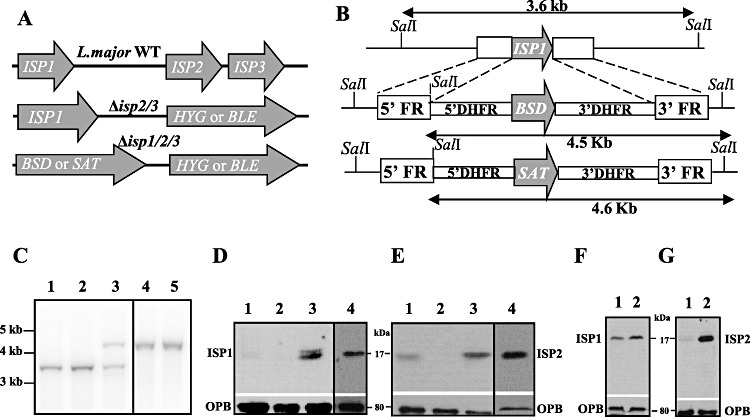
Generation of *L. major* ISP1, ISP2 and ISP3 triple null mutants. A. Schematic representation of the *ISP* loci of *L. major* in wild-type (WT, upper), Δ*isp2/3* (middle) and Δ*isp1/2/3* (lower) parasites. B. Schematic representation of the *ISP1* locus in WT *L. major* (upper) and the constructs for gene deletion. ORFs are shown as grey arrows and 5′ and 3′ flanking regions (FR) as boxes representing the DNA sequences used for targeting. The predicted fragment sizes after restriction digest are shown. *BSD*, blasticidin resistance gene; *SAT*, nourseothricin resistance gene. C. Southern blot of WT *L. major* (lane 1), Δ*isp2/3* (lane 2), first allele ISP1 knockout heterozygous Δ*isp1/2/3* (lane 3) and two Δ*isp1/2/3* clones (lanes 4 and 5) digested with SalI and probed with radiolabelled 5′ FR. D. Western blots of cell extracts from 1 × 10^7^ stationary-phase promastigotes of WT *L. major* (lane 1), Δ*isp1/2/3* (lane 2), Δ*isp1/2/3:*ISP1 (lane 3), Δ*isp1/2/3:ISP2-3*[*pXG-ISP1*] (lane 4), using purified αISP1 primary antibody raised in sheep. E. Western blot of cell extracts from 1 × 10^7^ stationary-phase promastigotes of WT *L. major* (lane 1), Δ*isp1/2/3* (lane 2), Δ*isp1/2/3:ISP2-3* (lane 3), Δ*isp1/2/3:ISP2-3*[*pXG-ISP1*] (lane 4) using purified α-ISP2 primary antibody raised in sheep. F. Western blot of cell extracts from 1 × 10^7^ stationary-phase promastigotes of WT *L. major* (lane 1) and WT [*pXG-ISP1*] (lane 2). G. Western blot of cell extracts from 1 × 10^7^ stationary-phase promastigotes of WT *L. major* (lane 1) and WT [*pXG-ISP2*] (lane 2). Antibodies to Oligopeptidase B (OPB) were used as loading control.

*Leishmania major ISP1*–*ISP2*–*ISP3* triple null mutants (Δ*isp1/2/3*) were created by sequential removal of two *ISP1* alleles from the previously described Δ*isp2/3* parasites (Eschenlauer *et al*., 2009) (Fig. 1A and B). Southern blotting confirmed the removal of both *ISP1* alleles, as the 3.6 kb WT alleles detected with a 5′ flank probe on SalI digested genomic DNA (Fig. 1C, lane 1) were present in Δ*isp2/3* (lane 2), while one allele was replaced with a drug resistance marker in heterozygous parasites (lane 3) and both replaced in Δ*isp1/2/3* parasites (lanes 4 and 5). *ISP1* was re-introduced into the ribosomal locus of Δ*isp1/2/3* to generate Δ*isp1/2/3:ISP1* and ISP2 and ISP3 were likewise re-introduced to generate Δ*isp1/2/3:ISP2-3.* A third re-expressing cell line Δ*isp1/2/3:ISP2-3*[*pXG-ISP1*] was generated by transfecting an episomal copy of *ISP1* into Δ*isp1/2/3:ISP2-3* parasites. Additionally, episomal plasmids for *ISP1* and *ISP2* were transfected into WT *L. major* to generate overexpression lines WT [*pXG-ISP1*] and WT [*pXG-ISP2*] respectively. Western blotting confirmed that ISP1 (Fig. 1D, lane 2) and ISP2 (Fig. 1E, lane 2) were absent from Δ*isp1/2/3* parasites and that ISP1 and ISP2 were expressed in all the add-back and overexpression cell lines (Fig. 1D–G).

### ISP1 has low peptidase inhibitory activity and is not involved in the uptake and initial survival of *L. major* in macrophages

**Fig. 2:**
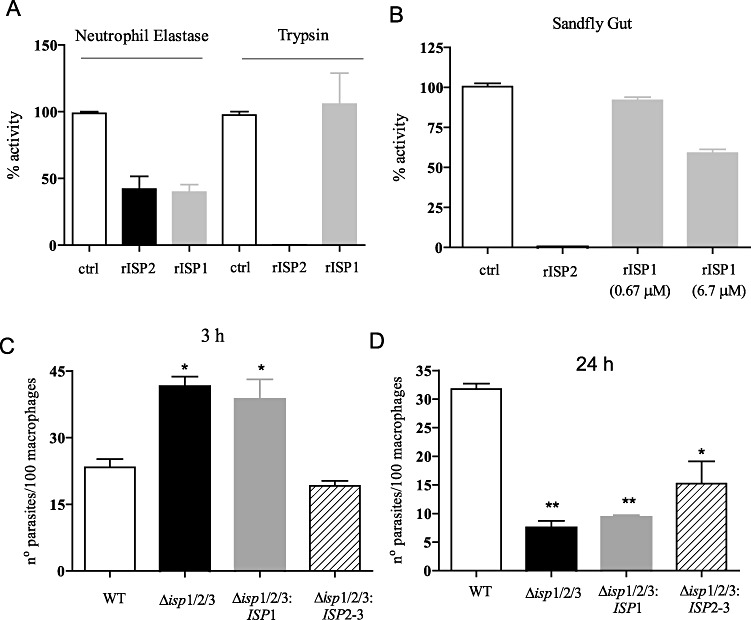
No role for ISP1 in uptake and survival of *L. major* in macrophages A. Inhibition of serine peptidases by recombinant ISPs. Human neutrophil elastase or bovine trypsin were incubated with rISP2 (200 nM) or rISP1 (670 nM) in 100 mM Tris-HCl pH 8, for 20 min on ice, and the residual peptidase activity was determined using MeOSuc-AAPV-AMC or Z-R-AMC as substrates respectively. B. Extracts from the midgut of *Phlebotomus papatasi* were incubated with rISP2 (200 nM) or rISP1 in 100 mM Tris-HCl pH 8, 100 mM NaCl, for 5 min on ice and the residual peptidase activity was determined using Boc-LGR-AMC as substrate. The graphs show average ± SEM of duplicates and are representative of two independent experiments. C. Number of parasites/100 macrophages was determined following incubation of stationary-phase promastigotes with elicited peritoneal macrophages from BALB/c mice at a 5:1 parasite : macrophage ratio for 3 h. D. Extracellular parasites were removed after 3 h interaction and the cells were cultured for 24 h to evaluate intracellular survival of amastigotes. The experiments were performed in triplicate, two independent times. The asterisk indicates statistical significance at *P* < 0.05 in relation to wild type.

To determine whether ISP1 has a role in modulating mammalian host serine peptidase activity and thereby influencing uptake and survival within murine macrophages, as has been shown for ISP2 (Eschenlauer *et al*., 2009), activity and infection assays were performed. Recombinant ISP1 was produced in *E. coli,* purified using affinity and ion-exchange chromatography and tested for inhibitory activity against mammalian clan PA, family S1A serine peptidases. ISP1 inhibited approximately 50% of human NE activity when pre-incubated with the enzyme at 80-fold excess, while ISP2 showed approximately the same degree of inhibition at 20-fold excess (Fig. 2A). The determination of the inactivation constant revealed a *K*_i_ of 107 ± 22 nM using MeOSuc-AAPV-AMC as a substrate. This was 10-fold higher than ISP2 assayed under the same conditions(*K*_i_ 8.8 ± 2.0 nM). ISP1 did not inhibit bovine trypsin, even when tested at 10 µM, in contrast to ISP2, which inhibited the enzyme completely at 2 µM and gave 50% inhibition at 0.2 µM (Fig. 2A). These results suggest that ISP1 is more selective than ISP2 and is a less potent inhibitor of these representative peptidases. Next, the inhibitory activity against serine peptidases present in the invertebrate host *Phlebotomus papatasi* was evaluated using extracts of the sand fly midgut as a source of peptidases (Fig. 2B). We observed full inhibition of the measured peptidase activity by ISP2 at 0.2 µM while ISP1 at 6.7 µM gave approximately 40% inhibition, showing that while both ISPs are capable of modulating the activity of sand fly midgut peptidases, ISP2 is the more efficacious.

Δ*isp1/2/3* parasites were used to infect elicited BALB/c macrophages in parallel with the cell lines re-expressing ISP1 or ISP2 (Δ*isp1/2/3:ISP1* and Δ*isp1/2/3:ISP2-3*). Δ*isp1/2/3* promastigotes were internalized twofold more efficiently than WT and the phenotype was compensated in the Δ*isp1/2/3:ISP2-3* but not the Δ*isp1/2/3:ISP1* parasites (Fig. 2C). The survival of Δ*isp1/2/3* parasites was severely reduced at 24 h and the phenotype was partly reversed in the Δ*isp1/2/3:ISP2-3*, but not Δ*isp1/2/3:ISP1* parasites (Fig. 2D). These experiments show that whereas ISP2 influences the uptake and initial survival of *L. major* in macrophages, ISP1 does not.

### *Δisp1/2/3* parasite populations have a growth defect and contain aggregates of cells with often tortuous morphology

**Fig. 3:**
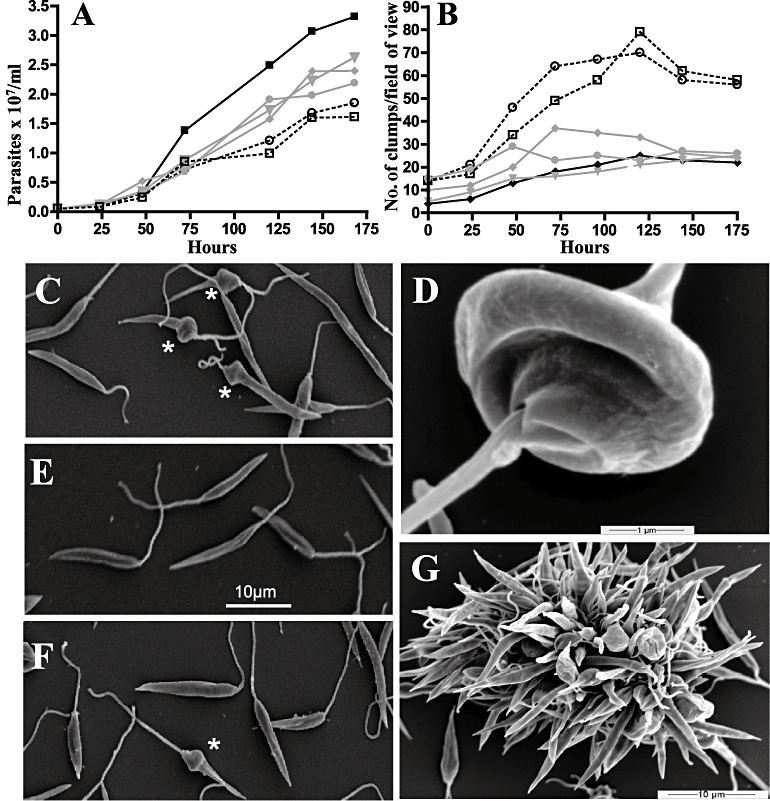
Growth and appearance of Δ*isp1/2/3* parasite populations A. Growth curve showing WT *L. major* (black squares), Δ*isp1/2/3*, two independent clones (black dashed, squares and circles) and Δ*isp1/2/3:ISP1,*Δ*isp1/2/3:ISP2-3,*Δ*isp1/2/3:ISP2-3*[*pXG-ISP1*] parasites (grey triangle, diamond and circle respectively). B. Formation of clumps as determined by light microscopy in 10 fields of view (×40 objective) in six-well plates. (A) and (B) were co-determined and the graphs are representative of four independent experiments. C, E and F. SEM of Δ*isp1/2/3*, Δ*isp1/2/3:ISP1* and Δ*isp1/2/3:ISP2-3*, the scale bar in (E) is applicable to all three images. Misshapen cells in (C) and (F) are denoted by a white asterisk. D. Anterior view of Δ*isp1/2/3* distended flagellar pocket. G. SEM of typical Δ*isp1/2/3* aggregate.

Given the lack of trypsin-fold serine peptidases in the *L. major* genome, we had anticipated that the ISPs would likely have an extracellular role that involved inhibition of host enzymes. The triple null mutant promastigotes, however, displayed a distinct growth phenotype. Cultured Δ*isp1/2/3* parasites grew normally during early log phase (up to 2 days) but, by mid-log phase, had a decreased rate of growth and reached stationary phase slightly earlier and at half the culture density of WT parasites (Fig. 3A). This was partially alleviated in *ISP1*, *ISP2-3* and the *ISP1-2-3* add-back cell lines. In conjunction with a reduced growth rate, about 30% of the parasites in the two clonal Δ*isp1/2/3* populations had unusual morphology, characterized by an enlargement of the flagellar pocket region (Fig. 3C, white asterisk and Fig. 3D, at higher magnitude). Concomitantly with the decrease in the growth rate (at approximately 48 h), the Δ*isp1/2/3* cultures contained unusually large parasite aggregates (Fig. 3B), formed from high numbers of often misshapen cells aggregating with their anterior pole at the centre of the mass (Fig. 3G). This was over and above the rosetting phenomenon commonly observed in late stage *L. major* cultures *in vitro*. As the growth cycle progressed, we observed increasing numbers of parasite aggregates in the Δ*isp1/2/3* cultures (Fig. 3B), but not in cultures of Δ*isp1/2/3:ISP1*. All the Δ*isp1/2/3:ISP1* parasites were of WT appearance (Fig. 3E) and formed fewer clumps, implicating the lack of *ISP1* in the abnormal parasite morphology and tendency to form aggregates. Aggregate formation was also abrogated in the Δ*isp1/2/3:ISP2-3* parasites, but abnormal cells with distended, twisted flagellar pockets were still apparent in the population at low numbers (Fig. 3F). These data suggest that ISP1 has a role in *L. major* promastigotes which is not complemented by ISP2-3.

### *Δisp1/2/3* parasite populations have longer flagella and are less motile than WT populations

**Fig. 4:**
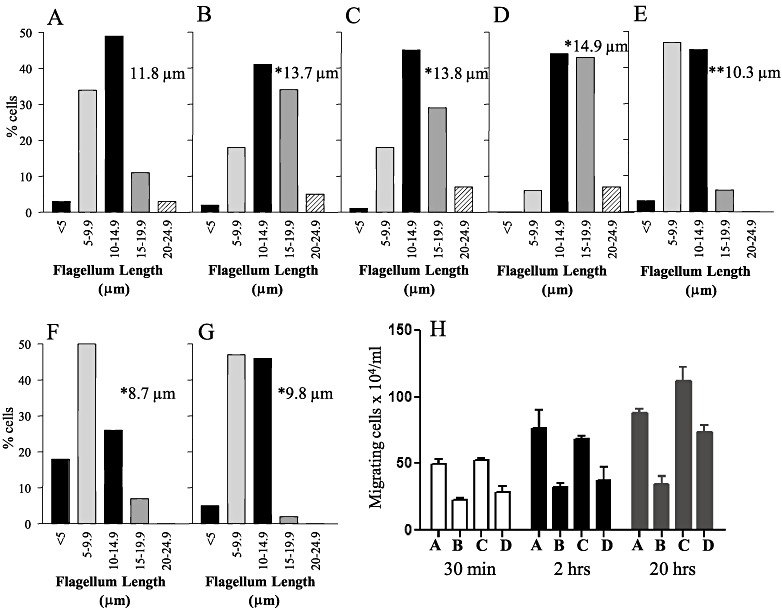
Δ*isp1/2/3* parasite populations have longer flagella and are less motile than WT populations. A–G. (A) WT *L. major*; (B) Δ*isp1/2/3*; (C) Δ*isp1/2/3:ISP1*; (D) Δ*isp1/2/3:ISP2-3*; (E) Δ*isp1/2/3:ISP2-3*[*pXG-ISP1*]; (F) WT [*pXG-ISP1*]; (G) WT [*pXG-ISP2*]. Grouped flagella length measurements expressed as a % of 200 flagella measured using iTEM software and ×550 SEM microscopy. Figures in grey shaded boxes are the mean flagella lengths for each graph and (*) or (**) denotes statistically difference at *P* < 0.001 or *P* < 0.01 of the entire data set from the WT (A), as measured by one-way anova with a Tukey post test. A full statistical analysis is shown in Table S1. H. Crossing assay showing the number of parasites (A, B, C and D as described above) crossing a 3.0 µM Transwell® membrane in 30 min (white bars), 2 h (black bars) and 20 h (grey bars). Error bars are SD of the mean for four replicates.

Given the identified distortion in the flagellar pocket region and that proteins orthologous to both ISP1 and ISP2 had been identified in the *T. brucei* flagellar proteome (Broadhead *et al*., 2006), we used scanning electron microscopy and iTEM software to measure flagella lengths in WT and mutant parasites in late log cultures. Only cells not in aggregates were considered in the measurements. The mean length of a flagellum in an *L. major* WT population was 11.8 µm (Fig. 4A), while that of Δ*isp1/2/3* parasite populations was significantly higher at 13.7 µm (Fig. 4B, Table S1). Reintroduction of either *ISP1* or *ISP2*-*ISP3* alone did not revert the phenotype as Δ*isp1/2/3:ISP1* and Δ*isp1/2/3:ISP2-3* parasites had mean flagella lengths of 13.8 µm and 14.9 µm respectively (Fig. 4C and D). The triple add-back parasites Δ*isp1/2/3:ISP2-3*[*pXG-ISP1*]*,* however, had a mean flagellum length of 10.3 µm (Fig. 4E), which was significantly lower than that of WT cells (Table S1), suggesting that the presence of all three *ISP* genes is required for flagellar homeostasis and so correct flagellar length. A more detailed analysis of the flagella lengths, whereby they were classified into length groupings of 0–4.9 µm, 5–9.9 µm, 10–14.9 µm, 15–19.9 µm or 20–24.9 µm revealed that the increased flagella length of the Δ*isp1/2/3,*Δ*isp1/2/3:ISP1* and Δ*isp1/2/3:ISP2-3* populations was due to a shift from the submedian 5–9.9 µm to the post-median 15–19.9 µm grouping in all these genotypes. The reverse was true in Δ*isp1/2/3:ISP2-3*[*pXG-ISP1*] parasites accounting for their shorter mean flagella length. We postulated that this may be due to excess ISP1. To address this, we determined the mean flagellum lengths of WT [*pXG-ISP1*] and WT [*pXG-ISP2*] to be 8.7 µm and 9.7 µm respectively (Fig. 4F and G). The data show that overexpression of either ISP1 or ISP2, in the presence of endogenous levels of ISP1 and ISP2, results in a reduction in mean flagellum length. As ISP1 was expressed from a multicopy plasmid in Δ*isp1/2/3:ISP2-3*[*pXG-ISP1*] and ISP2 was previously identified as overexpressed in the ribosomal locus of Δ*isp2/3:ISP2-3* (Eschenlauer *et al*., 2009), the results generally show that overexpression of either ISP in the triple add-back parasites could account for their short flagellum.

We observed that a great number of the Δ*isp1/2/3* parasites in the cultures with no apparent morphological abnormalities displayed altered movement. We investigated this by testing the ability of un-aggregated promastigotes to cross pored membranes in Transwell chambers. Reduced motility was observed, Δ*isp1/2/3* parasites were found to have crossed a membrane with only half the efficiency of WT parasites at each of the three time points studied (Fig. 4H). This was fully compensated in the Δ*isp1/2/3:ISP1* parasites but not the Δ*isp1/2/3:ISP2-3* parasites, which only showed a motility equivalent to that of WT at the 20 h time point. Taken together, these data suggest that the balance between the levels of the different ISPs is important for the maintenance of flagellum length and function.

### Increased abundance of haptomonad promastigotes in *Δisp1/2/3* parasite populations

**Fig. 5:**
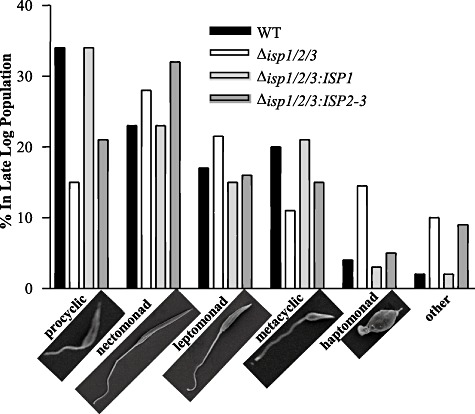
Differentiation of Δ*isp1/2/3.* Parasites in late log phase cultures were examined at ×550 magnification using SEM and classified into the morphological categories for promastigotes as previously described (Rogers *et al*., 2002). The experiment was performed with duplicate SEM samples independently processed and the graph is typical of 200 parasites in each population.

Flagellum length, particularly in relation to cell body size and morphology, is a distinguishing feature in different promastigote life cycle stages, so we quantified, based on both morphology (Fig. 5) and flagellum length, the numbers of procyclic, nectomonad, leptomonad, haptomonad and metacyclic promastigotes present in late log phase cultures. WT populations contained more than twice as many procyclic promastigotes than Δ*isp1/2/3* populations. This difference was also apparent in Δ*isp1/2/3:ISP2-3* populations, but Δ*isp1/2/3:ISP1* populations contained as many procyclic promastigotes as WT populations; thus ISP1 complemented the phenotype but ISP2 did not. While slightly increased numbers of nectomonad and leptomonad promastigotes in the Δ*isp1/2/3* population partially accounted for the decrease in procyclic promastigotes, the most marked increase was in the number of haptomonad parasites. In addition, the WT populations had nearly twice the number of metacyclic promastigotes than the Δ*isp1/2/3* populations, a change that was also compensated by ISP1 but not ISP2 (Fig. 5). While unclassified cells also increased in the Δ*isp1/2/3* populations, the major differentiation shift in Δ*isp1/2/3* populations was due to the decrease in procyclic and metacyclic promastigotes and an increase in the intermediate stages with predominance towards haptomonad promastigotes.

### ISP1 and ISP2 localize to the flagellar collar in haptomonad promastigotes

**Fig. 6:**
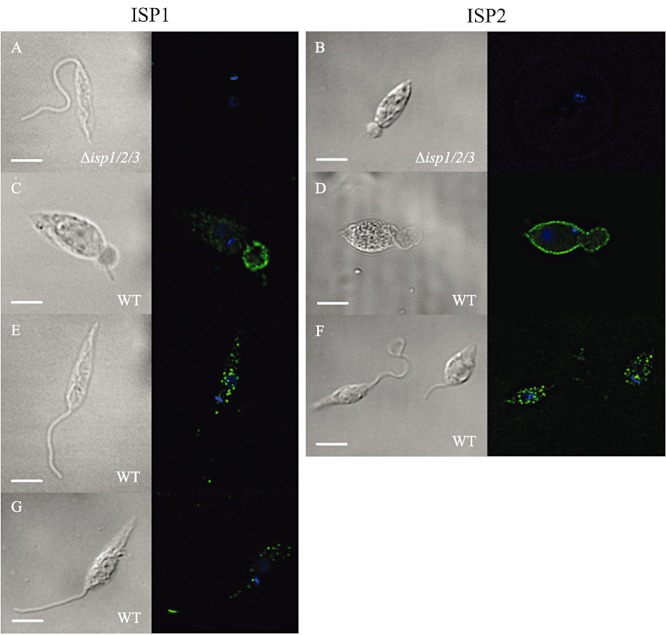
ISP1 and ISP2 localization. Fixed *L. major* were labelled with anti-ISP1 (A, C, E and G) or anti-ISP2 (B, D and F) antibody and donkey anti-sheep Alexa 488-secondary antibody. Nuclear and kinetoplast DNA were stained with DAPI. Left panel, DIC image. Right panel, merged image of ISP (green) and DAPI (blue). Scale bar = 5 µm.

We purified polyclonal anti-ISP1 and anti-ISP2 antibodies to a high specificity, and neither was found to have cross-reactivity with Δ*isp1/2/3* parasites in fluorescence microscopy (Fig. 6A and B). Both ISP1 and ISP2 showed a prominent labelling of the hemidesmosome in haptomonad promastigotes, where a very strong signal was detected (Fig. 6C and D), in addition to a cell surface localization for ISP2. In other promastigote life cycle stages, ISP1 and ISP2 were found in the cytosol as well as in distinct foci along the flagellum, with a noticeable accumulation at the tip of the flagellum in approximately 70% of cells analysed (Fig. 6E–G).

ISP1 and ISP2 orthologues were absent from the *T. brucei* flagellar proteome performed on *snl*-*1* RNAi mutants (Hart *et al*., 2009), although they had previously been identified in this organelle in WT *T. brucei* (Broadhead *et al*., 2006) (Table S2). The *snl*-*1* mutants are severely deficient in the PFR protein, PFR-A, and lack the intermediate and distal regions of the PFR which results in parasite paralysis (Bastin *et al*., 1999). An equivalent *snl-1* mutant has been generated in *Leishmania mexicana* by deletion of the *PFR2* gene (Santrich *et al*., 1997). We therefore aimed to determine the localization of ISP1 and ISP2 in WT *L. mexicana* and in Δ*pfr1* and Δ*pfr2* mutants. Western blot analysis showed that ISP1 could be detected in WT *L. mexicana* parasites and that expression was significantly reduced in both Δ*pfr1* and Δ*pfr2* mutants (Fig. S1A), a finding that correlates with the proteomics analysis of the *T. brucei snl-1* mutants*.* ISP1 had the same subcellular localization in both *L. major* and *L. mexicana,* including the hemidesmosome of haptomonads but was absent from the flagellum of Δ*pfr1* and Δ*pfr2 L. mexicana* (Fig. S1B)*.* Similar analyses for ISP2 were not possible as antibody raised against *L. major* ISP2 did not detect *L. mexicana* ISP2 in either Western blots or immunofluorescence (Fig. S1C and D)*,* despite the presence of an intact gene in *L. mexicana* (Rogers *et al*., 2011).

### Excessive vesicles and membraneous whorls in the flagellar pockets of *Δisp1/2/3* parasites

**Fig. 7:**
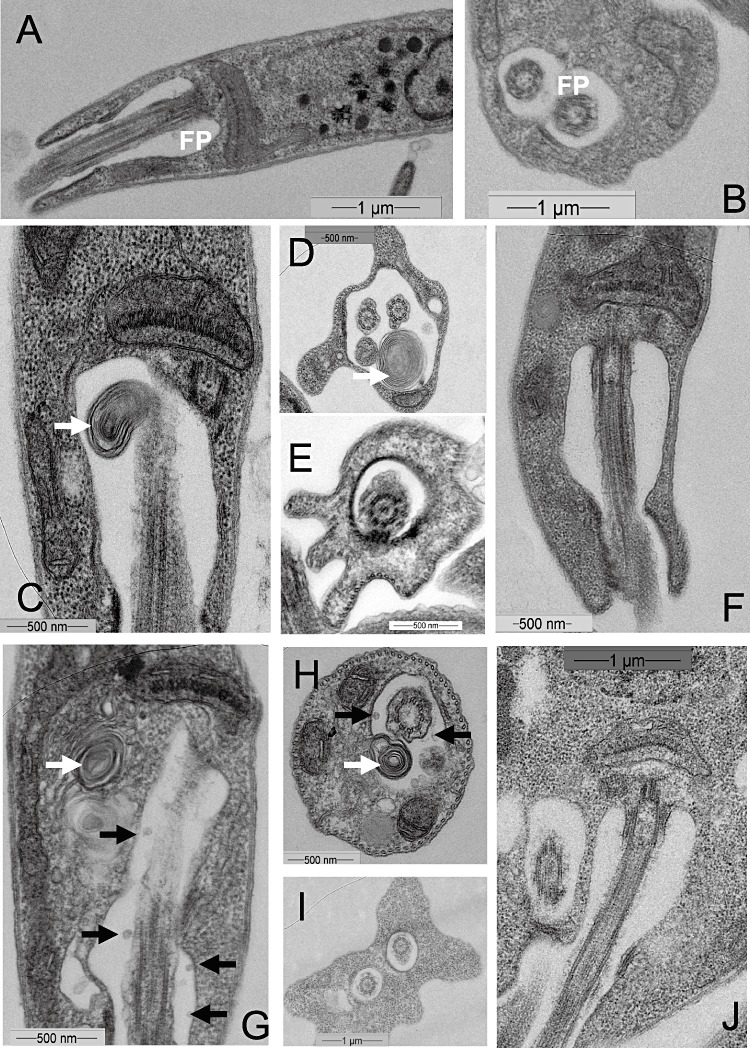
Flagellar pocket structure in Δ*isp1/2/3* parasites. Transmission EM images of WT (A, B), Δ*isp1/2/3* (C, D), Δ*isp1/2/3*:ISP1 (E, F), Δ*isp1/2/3*:ISP2:3 (G, H) and Δ*isp1/2/3:ISP2-3*[*pXG-ISP1*] (I, J) promastigote flagellar pockets (FP). Membranous whorls in Δ*isp1/2/3* (C, D) and Δ*isp1/2/3*:ISP2-3 (G, H) are indicated with a white arrow and small vesicles in Δ*isp1/2/3*:ISP2-3 (G, H) with a black arrow. FP flagellar pocket, DG dense granule.

To identify the cause of the distortion in the flagellar pocket of Δ*isp1/2/3,* we examined longitudinal and cross-sections of the flagellar pocket region by transmission electron microscopy (TEM). WT parasites (Fig. 7A and B) have largely clear unobstructed flagellar pockets. The Δ*isp1/2/3* parasites, however, accumulated dense granular vesicles and membranous whorls (Fig. 7C and D). Release of this material into the flagellar pocket was not observed in the Δ*isp1/2/3:ISP1* (Fig. 7E and F) or Δ*isp1/2/3:ISP2-3*[*pXG-ISP1*] (Fig. 7I and J), whereas vesicles and whorls were detected in the Δ*isp1/2/3:ISP2-3* parasites (Fig. 7G and H). The Δ*isp1/2/3* parasites have a normal 9+2 axoneme and PFR (Fig. 7D). This demonstrates that loss of ISP1, but not ISP2, causes a disruption in membrane dynamics within the flagellar pocket but does not affect the structure of the flagellum.

### Excessive secretion from *Δisp1/2/3* parasites

**Fig. 8:**
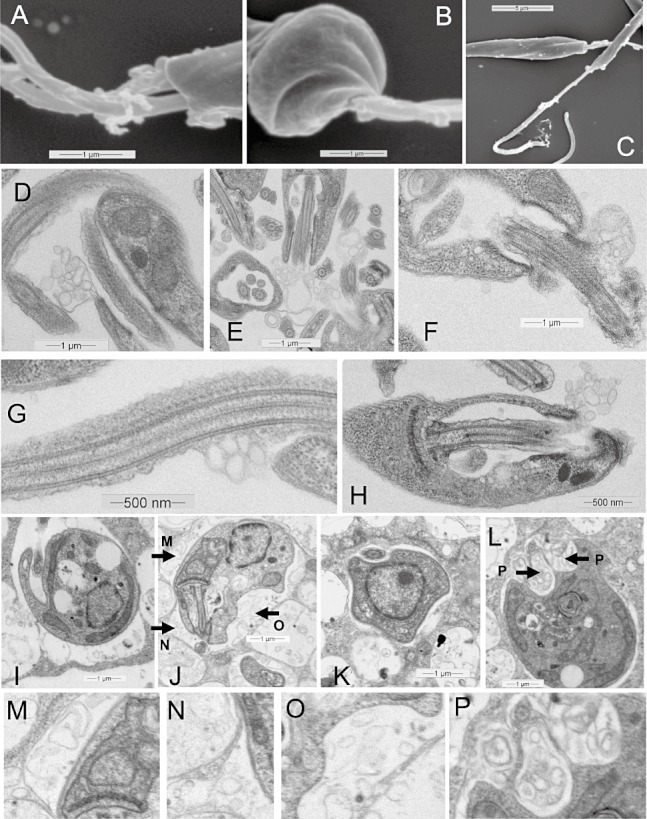
Release of membrane vesicles in Δ*isp1/2/3.* A–C. SEM of Δ*isp1/2/3* parasites. D–H. TEM of Δ*isp1/2/3*. I–L. RAW macrophage infected with (I) WT *L. major*, (J) Δ*isp1/2/3*, (K) Δ*isp1/2/3:ISP1*, (L) Δ*isp1/2/3:ISP2-3*. Membrane vesicles released in (J) and (L) are indicated with black arrows and magnified in (M)–(P).

Scanning electron microscopy (SEM) and TEM also revealed that Δ*isp1/2/3* promastigotes secreted/excreted excess material from their anterior poles. SEM showed that the material was emerging from the flagellar pocket (Fig. 8A–C) and in some cases appeared to slough down the flagellum and shed from the tip (Fig. 8C). TEM gave a more detailed picture of the exudation, and material was visualized emitting from the flagellar pocket, at the hemidesmosome (Fig. 8D and E) and apparently budding from the flagellum itself (Fig. 8F–H). While some dense granules could be seen, the majority of the material released from the Δ*isp1/2/3* parasites comprised relatively transparent, apparently membrane-bound vesicles of ∼ 100 nm diameter. These vesicles were found in regions of TEM sections with a concentration of flagellum (Fig. 8E), but this was not exclusive (Fig. 8D and F–H). It is also apparent that, while the exuded vesicles are clearly membrane-bound as they leave the parasite, their membrane integrity diminishes as they dissipate from the point of release (Fig. 8D–H). A similar release of membrane material was observed in Δ*isp1/2/3* (Fig. 8J, arrowed and in higher magnification in 8M–O) and Δ*isp1/2/3:ISP1* (Fig. 8L, arrowed and in higher magnification in 8P) intracellular amastigotes within macrophages but not in WT (Fig. 8I) or Δ*isp1/2/3:ISP2-3* (Fig. 8K).

## Discussion

*Leishmania major* ISP1 and ISP2 share only 36% sequence identify with each other and have very distinct inhibitory properties. While rISP2 is a relatively potent inhibitor of all the clan PA, family S1A serine peptidases tested so far (Eschenlauer *et al.*, 2009), the inhibitory activity of rISP1 is more restricted and is at least 10-fold lower than that of rISP2. The interaction of bacterial ecotins with the active site of serine peptidases occurs via the primary binding site composed of the 50s and 80s loops, the latter bears the reactive Met84 residue (Yang *et al*., 1998). While the residues composing the 80s loop of ISP1 have similarity to those of ecotins (more so than ISP2), ISP1 lacks the reactive Met which would be predicted to influence its inhibitory capacity. Furthermore, the tripeptide that comprises the putative 50s loop of ISP1 (HQT) has no conservation with ecotin (LHR), whereas ISP2 (VYR) bears conserved substitutions in those positions. Thus ISP1 and ISP2 appear to present significant differences in the binding site to target enzymes, which are consistent with, and suggestive of, important functional diversity. This diversity must reside within the ecotin-binding domain as the predicted molecular weight of ISP1 is 16.4 kDa and the ecotin domain (Pfam domain: PF03974) covers 95% of the protein. Our results indeed show that the two natural inhibitors have different inhibitory specificities, with rISP2 inhibiting NE and trypsin with Kis in the low nM range, whereas rISP1 is less active against NE and has no inhibitory activity towards trypsin. ISP2, but not ISP1, has been demonstrated to prevent TLR4 activation through inhibition of mammalian NE, thus promoting survival in macrophages (Eschenlauer *et al*., 2009; Faria *et al*., 2011). The demonstration that rISP1 is 10-fold less potent than ISP2 as an inhibitor of NE, possibly accounts for the observed lack of effect of ISP1 on macrophage invasion.

ISP1 is predominantly expressed in promastigotes, so a role in the inhibition of sand fly gut serine peptidases could be predicted. Sand flies express an abundance of trypsin-like and chymotrypsin-like serine peptidases and *Leishmania* must withstand the hydrolytic environment of the sand fly gut. Moreover, various *Leishmania* species have demonstrable trypsin-modulating activity *in vivo* (Dillon and Lane, 1993; Sant'Anna *et al*., 2009; Telleria *et al*., 2010; Dostalova *et al*., 2011). In this study, however, we found that whereas rISP2 was able to inhibit the serine peptidase activity of a sand fly midgut extract (showing 60% inhibition at 2 nM), under the same conditions rISP1 inhibited with less potency (with 40% inhibition at 6.7 µM). Thus these results reinforce the concept that ISP1 is less active against trypsin-like peptidases than ISP2, although it cannot yet be ruled out that ISP2 and ISP1 have functions that target different peptidases in the sand fly.

The current study has revealed, however, that ISP1 has an intracellular role within the parasite itself, which we did not anticipate as *L. major* apparently lacks endogenous S1A enzymes. Nevertheless, the distinctive *in vitro* phenotype discovered for Δ*isp1/2/3* is strongly suggestive of an intracellular role for ISP1, either targeting an as yet unidentified non-family S1 peptidase or a role that is independent of the inhibition of serine peptidase activity. The increased flagella length in Δ*isp1/2/3* parasite cultures may be explained in part by a change in the balance between the procyclic, nectomonad and leptomonad promastigote forms that occur during differentiation. Our detailed analysis of late log phase promastigote populations showed that Δ*isp1/2/3* cultures did vary from WT populations in containing more forms intermediate in the procyclic–metacyclic transformation. Potentially relevant to this, the intracellular distribution of both ISP1 and ISP2 also varied with promastigote differentiation. ISP1 and ISP2 in haptomonad promastigotes were found to be prominently located in the hemidesmosome (Fig. 6), a structure that is involved in attachment to the chitinous parts of the stomodeal valve (Killick-Kendrick *et al*., 1974b; Molyneux and Killick-Kendrick, 1987), although ISP2 also had a surface location. Parasite attachment results in destruction of the chitin lining and unique filaments on the apical end of cylindrical cells of the valve (Volf *et al*., 2004). These changes, together with the plug formed from the promastigote secretory gel, facilitate the regurgitation of *Leishmania* into the vertebrate host (Rogers *et al*., 2004; Volf *et al*., 2004). With this location, both ISP1 and ISP2 are well situated to interact with sand fly peptidases. In other promastigote forms the ISPs occur in the cytosol and in discreet foci at the tip of the flagellum. Localization to the flagellum is clearly dependent on a functional PFR, as ISP1 in Δ*pfr1* and Δ*pfr2* parasites was absent from the flagellum, which is consistent with proteomics analysis in trypanosomes (Broadhead *et al*., 2006; Hart *et al*., 2009).

The changes in the proportion of the different promastigotes forms in Δ*isp1/2/3* cultures cannot, however, explain fully the elongated flagellum phenotype in these parasites, because while re-expression of ISP1 alone restored the differentiation balance of the triple null mutant culture, the re-introduction of ISP2 was also required to complement fully the flagellum length phenotype. This suggests that whereas ISP1 is involved in promastigote differentiation, both ISP1 and ISP2 are required for flagellum homeostasis. The Δ*isp1/2/3* parasite flagella are not structurally disrupted, having a normal 9+2 microtubule arrangement and PFR, indicating that IFT is operating, but it is possible that the elongated flagella present in the Δ*isp1/2/3* parasites arise from disruption of the regulation of trafficking along the IFT rather than any loss of structural integrity itself. Alternatively, excess components not properly turned over during re-modelling and homeostasis that become accumulated in the flagellum itself could account for the alterations in flagellar length. It appears that ultimately some of the excess material is shed, based on the materials observed in the flagellar pocket region of the mutant promastigotes. It is not known how flagellar assembly is regulated in *Leishmania*, but mitogen kinases involved in the control of flagellar length by putative phosphorylation of flagellar components have been described (Wiese *et al*., 2003; Erdmann *et al*., 2006) and similar mechanisms have been proposed to modulate cargo recognition in a myosin-dependent flagellar protein trafficking pathway operating beside the IFT system (Katta *et al*., 2010). IFT, powered by kinesin and dynein motors, has an essential, if not solo, role in *Leishmania* flagella biosynthesis and silencing of either anterograde or retrograde transport results in shortening of the flagellum in trypanosomes (Absalon *et al*., 2008). This is similar to the phenotype seen in this study with the overexpression of ISP1 or ISP2 in *Leishmania* (Fig. 4). The ISP mutants characterized in the current study also have similar phenotypes to the *L. major* kinesin mutants, which have shortened flagella upon overexpression of kinesin, while comparable siRNA knock-down mutants of the equivalent *T. brucei* gene have elongated flagella due to the micro-tubule binding and depolymerizing activity of kinesin (Blaineau *et al*., 2007). Intriguingly, *Leishmania donovani* parasites lacking a component of the outer dynein arm docking complex (*LdDG2* null mutants) also demonstrated a link between flagellum length regulation and differentiation, and it was proposed that unincorporated flagellar proteins trigger stress responses which promote differentiation between parasite forms (Harder *et al*., 2010).

Another interesting feature of the Δ*isp1/2/3* parasites is the accumulation of vesicles and membranous whorls in their flagellar pocket. The accumulation of whorls was previously attributed to the breakdown of membranes during the shortening of the flagellum as parasites differentiated from the nectomonad to haptomonad life cycle stages (Killick-Kendrick *et al*., 1974a). This may account for this phenotype in the Δ*isp1/2/3* parasites cultures, as they contain more parasites undergoing differentiation; however, it does not explain the accumulation of large and small dense vesicles in the flagellar pockets, as previously reported in the *T. brucei* IFT mutants (Absalon *et al*., 2008). It therefore seems possible that the excessive accumulation of vesicles in the flagellar pockets of Δ*isp1/2/3* parasites is the result of a combination of altered differentiation and disruption of IFT, both of which are reversed by ISP1 but not ISP2.

The vesicles observed emerging from Δ*isp1/2/3* parasites were not confined to the flagellar pockets, but also occurred at the hemidesmosome where they surrounded the collars. The exudate does not appear to be the filamentous acid phosphatase known to be released from *Leishmania* promastigotes, as it is less fibrous in appearance (Stierhof *et al*., 1994). Rather, many of the vesicles secreted resemble in size and morphology those recently described as exosomes secreted from *L. donovani* (Silverman *et al*., 2010). Exosome secretion is a recent discovery in *Leishmania*, where the presence of an alternative secretory pathway has long since been accepted although elucidation of the mechanism and occurrence has been elusive (Stegmayer *et al*., 2005). However, unlike this recent *L. donovani* study, and a previous proteomic analysis of the *L. donovani* secretome (Silverman *et al*., 2008), we can see no evidence of exosome budding from the body of *L. major* WT or Δ*isp1/2/3* cells; however, we did detect significant release from the flagellum, flagellar pocket and hemidesmosome collar of the Δ*isp1/2/3* parasites which appear identical in appearance to the *L. donovani* exosomes and is not present in WT *L. major*. While it is unlikely that altered differentiation in the Δ*isp1/2/3* parasites results in upregulated release of vesicles via the exosome pathway, excessive secretion may arise as the parasites try to dispose of surplus or mis-trafficked intracellular material accumulating in their flagellar pockets due to erroneous flagellum homeostasis.

The findings of this study clearly show that ISP1 has an endogenous role in *Leishmania* promastigotes, although as yet its exact function in the parasite's homeostasis is not clear. We propose that ISP1 is involved in flagellum homeostasis and that its loss results in flagellar pocket distension. Together these culminate in excessive secretion and stress response-induced differentiation. This supports recent reports that the flagellum is a sensory organelle promoting morphological changes during trypanosomatid differentiation (Rotureau *et al*., 2009) and host–parasite interactions in amastigotes (Gluenz *et al*., 2010b). Further investigation of the Δ*isp1/2/3* parasites will aid understanding of flagellum homeostasis as well as the *Leishmania* exosomal secretory pathways.

## Experimental procedures

### Parasites

*Leishmania major* Friedlin (MHOM/JL/80/Friedlin) were grown as promastigotes in modified Eagle's medium (designated HOMEM medium) supplemented with 10% (v/v) heat-inactivated fetal calf serum (FCS) at 25°C as described previously (Besteiro *et al*., 2006). Gene deletion mutants were selected with 50 µg ml^−1^ hygromycin B (Roche), 20 µg ml^−1^ phleomycin (Invivogen), 10 µg ml^−1^ blasticidin (Merck), 100 µg ml^−1^ nourseothricin (Sigma) and 50 µg ml^−1^ puromycin (Calbiochem). 25 µg ml^−1^ G418 (Invitrogen) was used for re-expressing lines. Metacyclic promastigotes were isolated from stationary-phase culture by agglutination of promastigotes with peanut lectin as previously described (Sacks *et al*., 1985).

### Generation of *L. major* ISP mutants

*Leishmania major* triple ISP null mutants (designated Δ*isp1/2/3*) were generated from Δ*isp2/3* parasites (Eschenlauer *et al*., 2009). The two plasmids containing the antibiotic resistance cassettes used for the double allele deletion of *ISP1* were produced as follows. The 480 bp 5′ flanking region (FR) was generated by PCR with primers OL1434 (CGAAGCTTTAAGCACTTAAAGCCGTGGACG) and OL1435 (TAGTCGACGATGGGAATGTGGGTTCGGTTA). The 546 bp 3′ FR was generated with PCR primers OL1436 (TACCCGGGACATCTGCTTTCTAGCTCGCTC) and OL1437 (GCAGATCTGGTCAGTGTGGAGGTGAAGAGG). The PCR fragments were subcloned into pGEM-T and then released by restriction digest with HindIII/SalI for the 5′ FR and XmaI/BglII for the 3′ FR. The fragments were sequentially cloned into a similarly digested hygromycin-resistant plasmid pGL792 (Besteiro *et al*., 2004). To produce blasticidin- and nourseothricin-resistant plasmids, the hygromycin cassette was replaced with the SpeI/BamHI resistance cassettes from pGL 434 and pGL227 to give plasmids pGL1027 and pGL1028. The integration cassettes were digested from the plasmids with BglII/HindIII prior to transfection. For the re-expression of ISP1, a 440 bp PCR fragment containing *ISP1* ORF was amplified from *L. major* genomic DNA with the primers OL1474 (CGAGATCTTCATACTGCAAGATCGAGGCC) and OL1475 (ATGCGGCCGCCTCACTCCGTGGCTGCCTGCATC). The PCR fragment was subcloned into pGEM-T and the insert then cloned into the pRIB expression vector using BglII and NotI to give pGL1229. The re-expression construct for ISP2-ISP3 is pGL1005 as previously described (Eschenlauer *et al*., 2009). The integration cassettes from pGL1229 and pGL1005 were excised by digestion with PacI and PmeI before transfection. *L. major* promastigotes were electroporated and selected as previously described (Eschenlauer *et al*., 2009). To generate triple add-back parasites, Δ*isp1/2/3:ISP2-3* parasites were transfected with an episomal copy of *ISP1* (pGL1003) which was generated by amplifying *ISP1* from genomic DNA with the primers OL1457 (TACCCGGGATGTCATACTGCAAGATCGAG) and OL1316 (GCGGATCCTCATCCGTGGCTGCCTGCATC), digesting with XmaI/BamHI and ligating into the similarly digested pXG vector. The same construct was used to generate *pXG-ISP1*-overexpressing parasites by transfection into WT parasites. ISP2-overexpressing parasites (*pXG-ISP2*) were generated by amplifying *ISP2* from genomic DNA with the primers OL1458 (TACCCGGGATGTCCGACGCCGCTGGAAAG) and OL1318 (GCGGATCCTCACATCTCCCTTGCCTTGGTG) using the same methodology. Southern blots were performed as previously described (Eschenlauer *et al*., 2009).

### Macrophage infections

Macrophage infections were performed as previously described (Eschenlauer *et al*., 2009). Briefly, elicited peritoneal macrophages from BALB/c mice were infected at a 5:1 ratio with stationary-phase promastigotes, for 3 h in RPMI supplemented with 0.1% bovine serum albumin (BSA). For the survival assays, after 3 h the extracellular parasites were removed by extensive washing and the culture was placed at 37°C in RPMI-FCS medium overnight, before fixation and staining. RAW macrophages were infected at a 1:10 ratio with stationary-phase promastigotes and incubated at 37°C for 5 days prior to preparation for TEM microscopy. For each sample, 10^7^ macrophages were infected and the cells were gently lifted from flasks by the addition of cold PBS and gentle agitation prior to fixing as detailed below.

### Immunofluorescence analyses

For immunolocalization of ISP1 and ISP2, parasites and slides were prepared as previously described (Ambit *et al*., 2011). Freshly affinity-purified primary antibodies were used at a 1:50 (v/v) dilution in 0.1% (v/v) Triton X-100, 0.1% (w/v) BSA, in PBS (designated TB) and slides were incubated for 2 h at room temperature. The slides were washed and incubated with donkey anti-sheep Alexa Fluor 488-conjugated antibody (Invitrogen) at 1:2000 (v/v) in TB for 1 h at room temperature. VectaShield Mounting Medium with DAPI (Vector Laboratories) was then applied to the slide. Imaging was performed using a DeltaVision RT epifluorescent microscope under a ×100 objective and images were captured with a CoolSNAP HQ camera.

### Electron microscopy

For TEM, promastigotes were washed in PBS and resuspended in 250 mM trehalose. They were applied to a Formvar/carbon-coated grid and imaging was done at 120 kV in a Zeiss LEO 912 energy filtering transmission electron microscope at 30 eV. For sectioned parasites, promastigotes were fixed by adding a mixture of 2.5% (v/v) glutaraldehyde in 0.1 M phosphate buffer pH 7.4 for 40 min. Subsequent processing followed standard methods. Resin sections (100 nm thick) were zero-loss imaged with the Zeiss LEO 912 EFTEM. For SEM parasites were allowed to settle out from culture medium onto poly-l-lysine-coated glass coverslips then briefly rinsed in PBS to remove unattached cells, then fixed with 2.5% glutaraldehyde in 0.1 M cacodylate buffer. The attached parasites were then osmicated, acetone dehydrated and critical point dried from liquid CO_2_, sputter-coated with Au/Pd and imaged at 6 kV in a JSEM 6400.

### Crossing assay

Assays were performed in Transwell® Permeable Supports (Corning). The assays were performed in complete *Leishmania* growth-conditioned medium obtained from stationary-phase cultures of wild-type *L. major* (centrifuged at 1500 *g* to remove parasites and filtered using 0.2 µm syringe filters). Prior to the assay, 600 µl of filtered conditioned medium was added to each well and plates were allowed to equilibrate at 25°C for 1 h prior to addition of 100 µl of parasites at a density of 5 × 10^6^ ml^−1^ to the upper chamber. Following incubations of 30 min, 2 h and 20 h, the transwells were removed and the solution in the lower chamber was gently resuspended. The parasites were counted by placing aliquots of the homogenized solution in a haemocytometer.

### *K_i_* determination for recombinant ISP1

Expression and purification of recombinant *L. major* ISP1 (rISP1) has been described previously (Eschenlauer *et al*., 2009). *K*_i_ determination of rISP1 against human trypsin and NE (Calbiochem) was carried out as described previously for rISP2 (Eschenlauer *et al*., 2009). Briefly, 20 nM NE was pre-incubated with increasing concentrations of rISP1 (10 nM–2 µM) in 100 mM Tris-HCl pH 8.0, 2.5% (v/v) dimethyl sulfoxide (DMSO), for 20 min at 4°C. The reaction was initiated by addition of substrate (300 µM MeOSuc-AAPV-AMC) and enzyme substrate hydrolysis was monitored in a spectrofluorimeter in a continuous assay, by measuring the release of fluorescence (excitation 380 nm, emission at 460 nm). *K*_i_^app^ values were determined by non-linear regression with GraphPad Prism 5 using the Morrison equation for tight binding inhibitors to fit the curves and the *K*_i_ was calculated using the equation: *K*_i_ = (*K*_i_^app^[S])/([S] + *K*_m_), where the *K*_m_ of NE for MeOSuc-AAPV-AMC, determined experimentally, was 600 µM. Data are the mean ± standard deviation from three independent experiments. The effect of rISP1 on bovine trypsin (Sigma) was determined in a similar manner using 100 nM trypsin, 10 µM rISP1, 50 µM Bz-R-AMC, 2.5% (v/v) DMSO. The enzyme and rISP1 were pre-incubated for 40 min before starting the reaction. rISP2 was used as a positive control and gave 50% inhibition at 0.2 µM, as described previously (Eschenlauer *et al*., 2009).

### Inhibition of sand fly midgut peptidase activity by recombinant ISP1 and ISP2

Recombinant ISP1 and ISP2 (rISP1 and rISP2) were tested for inhibitory effects on the peptidase activity of extracts from the midgut of *P. papatasi*, a sand fly that is the natural vector for *L. major*. Sand flies were kept at 25°C in standard conditions as described by Volf and Volfova (2011) and dissected 48 h after bloodfeeding on a BALB/c mouse, when the protease activity in the midgut is at its maximal level. Pools of 10 guts in 100 µl of Tris buffer (20 mM Tris, 150 mM NaCl, pH 7.8) were homogenized, cleared by centrifugation (5000 *g*, 5 min) and stored at −70°C. The protein concentration of the soluble fraction was determined using the Bradford assay (Bio-Rad). The fly gut extract (0.75 µg^−1^) was pre-incubated with increasing concentrations of rISP1 (0.7 µM–7 µM) in 100 mM Tris-HCL pH 8.0, 100 mM NaCl, 2.5% (v/v) DMSO, for 5 min at 4°C. For ISP2, the fly gut extract (0.5 µg^−1^) was pre-incubated with increasing concentrations of rISP2 (2 nM–2 µM) in 100 mM Tris-HCl pH 8, 100 mM NaCl, for 5 min at 4°C. The reactions were initiated by addition of substrate (20 µM Boc-LGR-AMC) and the amount of AMC released was determined by monitoring fluorescence (excitation 380 nm, emission at 460 nm).

### Western blot analyses

Promastigote cultures (10^8^ cells) were centrifuged at 1000 *g* for 10 min, washed with PBS pH 7.2 and resuspended in 100 µl of SDS-PAGE sample buffer [45 mM Tris pH 6.8, 10% (v/v) glycerol, 1% (w/v) SDS, 0.01% bromophenol blue, 50 mM DTT]. The samples were vortexed then boiled for 5 min and 10 µl per sample was loaded on a 15% SDS-PAGE. Proteins were transferred onto Hybond C-Super nitrocellulose membranes (Amersham Pharmacia) and blocked in Tris-buffered saline (TBS) supplemented with 0.05% Tween-20 (TBST) and 5% (w/v) Marvel at 4°C overnight. Affinity-purified primary antibodies to either rISP1 or rISP2 were used at 1:50 (v/v) in TBST with 5% (w/v) Marvel, and purified anti-OPB antibodies were used at 1:20 000 (v/v) (Munday *et al*., 2011) for 2 h at room temperature. Secondary antibodies, donkey anti-sheep IgG-HRP (Santa Cruz Biotechnology) were used at a 1:10 000 dilution. The reactive bands were detected using ECL Western Blotting Detection Reagent (Amersham).

## Supporting information

**Fig. S1.** Analysis of ISP1 and ISP2 expressionin *L. mexicana*.

**Table S1.** Statistical analysis of mean flagellar length measurements.

**Table S2.** Flagellar proteins identified in WT but not snl2 RNAi mutant trypanosomes.
