# Supplementary material for: Ecotin-like serine peptidase inhibitor ISP1 of Leishmania major plays a role in flagellar pocket dynamics and promastigote differentiation
Source: Cell Microbiol. 2012 May 8;14(8):1271–86. doi: 10.1111/j.1462-5822.2012.01798.x (PMC3440592; doi:10.1111/j.1462-5822.2012.01798.x)
Supplement: Supplementary file 1 [file cmi0014-1271-SD1.pdf]

Supplementary Table 1

	A	B	C	D	E	F	G
**A. WT**		P<0.001	P<0.001	P<0.001	P<0.01	P<0.001	P<0.001
**B. Δ*isp1/2/3***	P<0.001		n/s	P<0.05	P<0.001	P<0.001	P<0.001
**C. Δ*isp1/2/3: ISP1***	P<0.001	n/s		n/s	P<0.001	P<0.001	P<0.001
**D. Δ*isp1/2/3: ISP2-3***	P<0.001	P<0.05	n/s		P<0.001	P<0.001	P<0.001
**E. Δ*isp1/2/3: ISP2-3* [*pxG-ISP1*]**	P<0.001	P<0.001	P<0.001	P<0.001		P<0.01	n/s
**F. WT [*pxG-ISP1*]**	P<0.001	P<0.001	P<0.001	P<0.001	P<0.01		n/s
**G. WT [*pxG-ISP2*]**	P<0.001	P<0.001	P<0.001	P<0.001	n/s	n/s	

Suppl. Table 1: *Δisp1/2/3 parasite populations have longer flagella and are less motile than WT populations* Statistical analysis of flagella lengths of *L. major* WT (A), Δ*isp1/2/3* (B), Δ*isp1/2/3:ISP1* (C), Δ*isp1/2/3:ISP2-3* (D), Δ*isp1/2/3:ISP2-3* [*pXG-ISP1*] (E), WT [*pXG-ISP1*] (F) and WT [*pXG-ISP2*] (G), as measured by one-way ANOVA with a Tukey post-test performed in GraphPad Prism 5.

Supplementary Figure 1

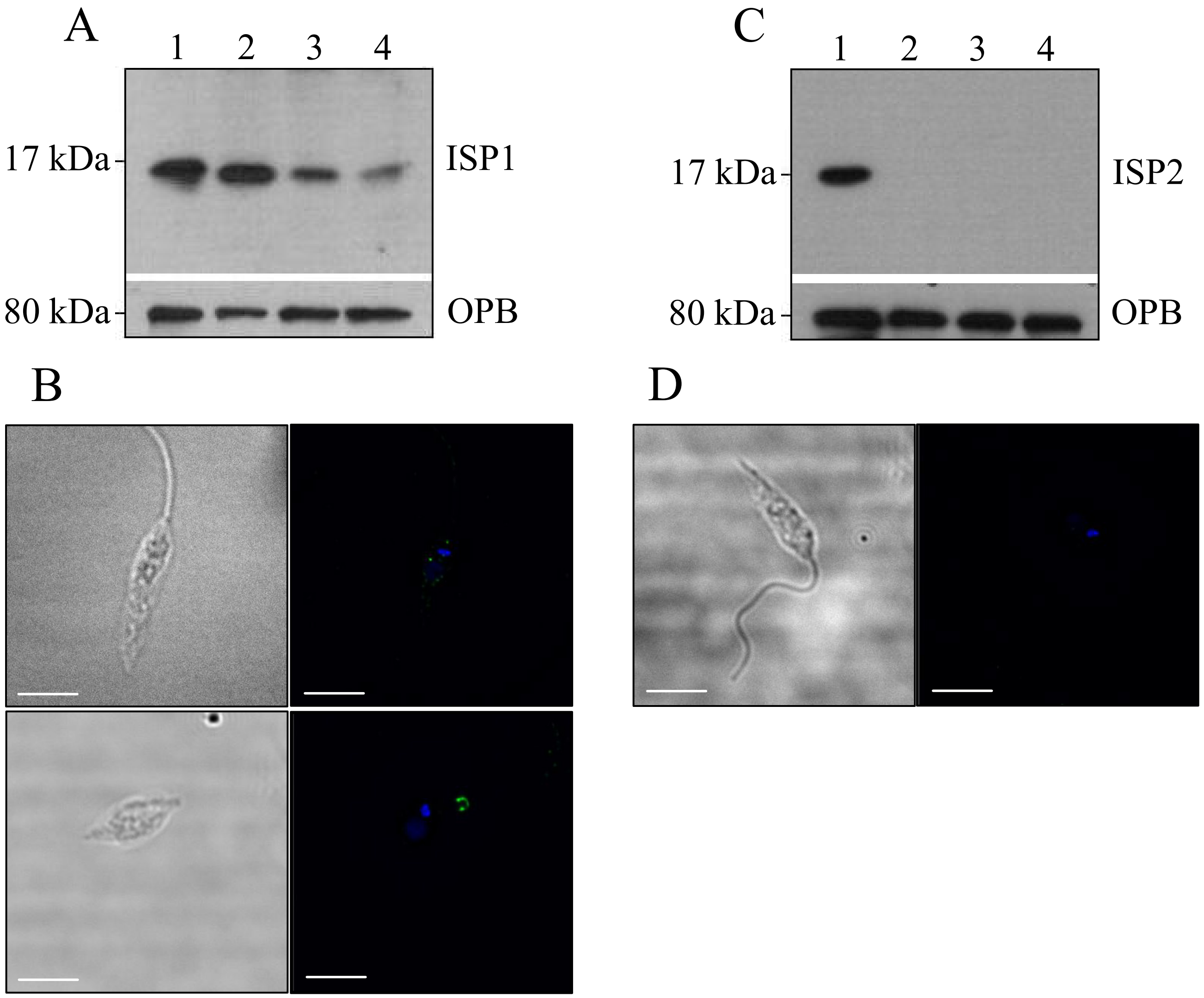

Suppl. Figure 1: Western blots of cell extracts from 1 x 10^7 stationary phase promastigotes of WT *L. major* (Lane 1), WT *L. mexicana* (Lane 2), *L. mexicana* Δ*pfr1* (Lane 3) and *L. mexicana* Δ*pfr2* (lane 4), using purified anti-ISP1 (A) or anti-ISP2 (C) primary antibody raised in sheep. Antibodies to Oligopeptidase B (OPB) were used as loading control. Fixed WT *L. mexicana* were labelled with anti-ISP1 (B) or anti-ISP2 (D) primary antibody and donkey anti-sheep Alexa 488-secondary antibody. Nuclear and kinetoplast DNA were stained with DAPI. Left panel, DIC image. Right panel, merged image of ISP (green) and DAPI (blue). Scale bar = 5 µm.
