# Supplementary figures and images for: Ecotin-like serine peptidase inhibitor ISP1 of Leishmania major plays a role in flagellar pocket dynamics and promastigote differentiation
Source: Cell Microbiol. 2012 May 8;14(8):1271–86. doi: 10.1111/j.1462-5822.2012.01798.x (PMC3440592; doi:10.1111/j.1462-5822.2012.01798.x)

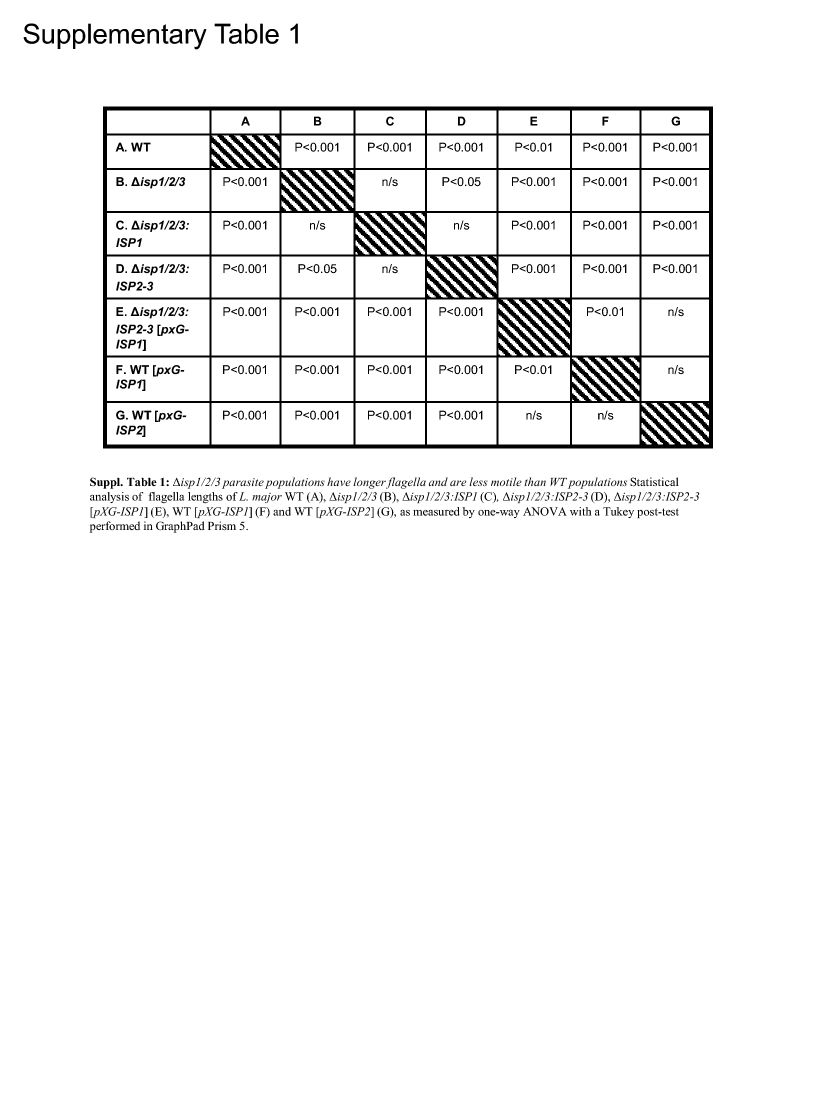

Supplement: Supplementary file 2 [file cmi0014-1271-SD3.png]
